# Supplementary material for: Systemic Sclerosis with Interstitial Lung Disease: Identification of Novel Immunogenetic Markers and Ethnic Specificity in Kazakh Patients
Source: Epidemiologia (Basel). 2025 Aug 6;6(3):41. doi: 10.3390/epidemiologia6030041 (PMC12372110; doi:10.3390/epidemiologia6030041)
Supplement: Supplementary file 1 [file epidemiologia-06-00041-s001.zip › epidemiologia-3732692-supplementary.pdf]

**SYSTEMIC SCLEROSIS WITH INTERSTITIAL LUNG DISEASE: IDENTIFICA-TION OF NOVEL IMMUNOGENETIC MARKERS AND ETHNIC SPECIFICITY IN KAZAKH PATIENTS**

**Supplement Table S1. Clinical and laboratory features of patients with SSc-ILD.**

| **№** | **Gender** | **Diagnosis** | **therapy** | | **RSS** | IL-6 | EScSG | ANA | Antibodies, Glow type | Genetic variants |
|---|---|---|---|---|---|---|---|---|---|---|
| | | | **steroids** | **cytostatics** | | | | | | |
| S1 | f | SSc, chronic course, diffuse form,generalized, activity 1 stage, with lesions of the skin (edema, hyper/depigmentation), joints (arthralgia, sclerodactyly), lungs(ILD), gastrointestinal tract, vessels (Raynaud's syndrome), liver (cirrhosis) | 8 | | 33 | 7,87 | 2 | 2560 | CENP-B, AC-3* centromere glow type | CD58(chr1:117078661 AT/A)-VUS<br>BANK1(chr4:102951224 GA/G) – VUS<br>SAMD9L(chr7:92764567 CA/C)-Likely pathogenic<br>LY96(chr8:74922341 CT/C) –VUS<br>PTPN22(chr1:114381166 CT/C) – VUS<br>IRAK1(chrX:153278833 GCC/GCCG) –VUS<br>IL2RA(chr10:6063607 A/C) –VUS<br>IL6R(chr1:154378136 GC/G) -Likely pathogenic<br>TREX1(chr3:48508185 T/TC) -Likely pathogenic |
| S3 | f | SSc, subacute course, diffuse form, with skin lesions ("purse-string" mouth, dense edema), joints (sclerodactyly, arthralgia), lungs (ILD), gastrointestinal tract (esophagitis), thyroid gland (hypothyroidism), blood vessels (Raynaud's syndrome) | 3 | D-penicillamine 250 mg | 9 | 1,33 | 1,5 | 320 | SS-A/52, core granular glow type (AC-4-5*). | CD58(chr1:117078661 AT/A)-VUS<br>SAMD9L(chr7:92762447 CA/C)-Likely pathogenic<br>LY96(chr8:74922341 CT/C) –VUS<br>DNASE1(chr16:3707747 G/A)-VUS<br>PTPN22(chr1:114381166 CT/C) – VUS<br>ITGA2B(chr17:42453072 G/GC (refGCC)) -Likely pathogenic |
| S5 | f | SSc, chronic course, diffuse form, generalized, activity 2 stage, skin lesions | 8 | | 13 | 3,7 | 3,5 | 5120 | CENP-B, AC-3* centromereglowtyp | BANK1(chr4:102951224 GA/G) – VUS |

| | | | | | | | | | | |
|---|---|---|---|---|---|---|---|---|---|---|
| | | (dense edema, depigmentation and hyperpigmentation, “purse-string” mouth), joints (arthralgia), lungs (ILD), gastrointestinal tract (dysphagia, esophagitis), thyroid gland (AITD), vessels (syndrome Raynaud's, telangiectasia).<br>Cushing’s steroid osteoporosis. | | | | | | | e | LY96(chr8:74922341 CT/C) –VUS<br>DNASE1(chr16:3707747 G/A)-VUS<br>PTPN22(chr1:114381166 CT/C) –VUS<br>IRAK1(chrX:153278833 GCC/GCCG) –VUS<br>AIRE(chr21:45711068 TC/T) -Likely pathogenic |
| S6 | f | SSc, chronic course, activity 2, withskinlesions(edema,induration, depigmentation), joints (polyarthritis),gastrointestinal tract (esophagitis),lungs (ILD,PAH, respiratory failure 2),secondarySjogren's syndrome. | 8 | nintedanib 200mg, leflunomide | 9 | 21,7 | 3,5 | 320 | Sm, SS-A/60, U1-snRNP, RNP/Sm, rib-P0 , Mixed: core granular glow type (AC-4-5*); AC-19* Cytoplasmic dense fine-granular pattern. Fluorescence is almost uniformly distributed throughout the cytoplasm. | CD58(chr1:117078661 AT/A)-VUS<br>BANK1(chr4:102951224 GA/G) –VUS<br>BANK1(chr4:102951316 GA/G) –VUS<br>SAMD9L(chr7:92762447 CA/C)-Likely pathogenic<br>SAMD9L(chr7:92764981 T/TT (реф TC))-Likely pathogenic<br>SAMD9L(chr7:92761606 GT/G)-Likely pathogenic<br>PTPN22(chr1:114381166 CT/C) –VUS<br>IRAK1(chrX:153278833 GCCCG/GCC) –VUS<br>RBPJ(chr4:26417097 GTTTTTTGC/GTTTTTTTG ref GTTTTTTTGC) –VUS<br>IL6R(chr1:154401686 G/A) -VUS |
| S9 | m | SSc, chronic course, diffuse form, activity 2, with damage to the lungs (ILD), skin (dense edema), joints (polyarthralgia), blood vessels (Raynaud's syndrome) | | nintedanib 150 mg | 15 | 2,33 | 5,5 | 0 | negative, AC-0* - negative | CD58(chr1:117078661 AT/A)-VUS<br>DNASE1(chr16:3707747 G/A)-VUS<br>PTPN22(chr1:114381166 CT/C) –VUS<br>CLEC16A(chr16:11260320 G/A) –VUS |

| | | | | | | | | | | |
|---|---|---|---|---|---|---|---|---|---|---|
| S10 | f | SSc, subacute course, diffuse form, generalized, activity 1, with lesions of the skin, joints, lungs (ILD), gastrointestinal tract (esophagitis), blood vessels (Raynaud's syndrome) | 4 мг | leflunomide 20 mg | 16 | **1,81** | 4,5 | 640 | CENP-B (Borderline result), core glow type (AC-8-10*). | CD58(chr1:117078661 AT/A)-VUS<br>BANK1(chr4:102951224 GA/G) – VUS<br>LY96(chr8:74922341 CT/C) –VUS<br>DNASE1(chr16:3707747 G/A)-VUS<br>PTPN22(chr1:114381166 CT/C) – VUS<br>ITGA2B(chr17:42455791 G/A) –VUS<br>AIRE(chr21:45708278 G/C) –VUS<br>RASGRP1(chr15:38800043 C/T) – VUS<br>RUNX1(chr21:36164691 G/T) –VUS<br>TLR2(chr4:154625677 GA/G) –VUS |
| S11 | f | SSc, generalized, stage 2, with lesions of the skin (dense edema), joints (polyarthralgia), gastrointestinal tract (esophagitis), lungs (ILD), blood vessels (Raynaud's syndrome), Sjogren's syndrome. | 4 мг | leflunomide 20 mg | 14 | 7,14 | 7 | 0 | negative, AC-0* - negative | CD58(chr1:117078661 AT/A)-VUS<br>SAMD9L(chr7:92762447 CA/C)-Likely pathogenic<br>LY96(chr8:74922341 CT/C) –VUS<br>DNASE1(chr16:3707747 G/A)-VUS<br>PTPN22(chr1:114381166 CT/C) – VUS<br>P2RX7(chr12:121603240C/T) –VUS |
| S12 | f | SSc, generalized, stage 2, with lesions of the skin, joints, lungs (ILD), blood vessels (Raynaud's syndrome), Sjogren's syndrome | | Methotrexat e 5 mg | 9 | 6,7 | 2 | 320 | SS-A/60,<br>SS-A/52,<br>SS-B,<br><br>CENP-B (Borderlineresult), core granular glow type (AC-4-5*). | CD58(chr1:117078661 AT/A)-VUS<br>BANK1(chr4:102951224 GA/G) – VUS<br>SAMD9L(chr7:92762447 CA/C)-Likely pathogenic<br>SAMD9L(chr7:92761606 GT/G)-Likely pathogenic<br>LY96(chr8:74922341 CT/C) –VUS<br>PTPN22(chr1:114381166 CT/C) – VUS |

| | | | | | | | | | |
|---|---|---|---|---|---|---|---|---|---|
| | | | | | | | | | AIRE(chr21:45708278 G/C) –VUS |
| S13 | f | SSc, generalized, stage 2, with lesions of the skin, joints, blood vessels (Raynaud's syndrome), lungs (ILD), gastrointestinal tract (reflux esophagitis) | | mycophenolate mofetil 1000mg. Leflunomide 20 mg | 23 | 26,3 | 5,5 | 160 | SS-A/60, SS-A/52, RNP/Sm, ribP0, U1-snRNP, ds-DNA, Nucleosome, Histon. Cytoplasmaticgranularglowtype (AC-19-20*). | CD58(chr1:117078661 AT/A)-VUS<br>SAMD9L(chr7:92762447 CA/C)-Likely pathogenic<br>STAT3(chr17:40467768 TG/T) –VUS<br>ARID5B(chr10:63852117 TA/T) –VUS<br>PTPRC(chr1:198682102 AT/A) -Likely pathogenic<br>SMAD3(chr15:67477130 AT/A) –Pathogenic<br>CD40(chr20:44750489 GA/G) -Likely pathogenic |
| S14 | f | SSc, generalized, with lesions of the skin, joints, blood vessels (Raynaud's syndrome), heart (cardiosclerosis), lungs (ILD), respiratory failure 2), gastrointestinal tract (esophagitis) | 4 | mycophenolate mofetil 1500mg | 18 | 2,15 | 7,5 | 1280 | SS-A/60, SS-A/52, CENP-B. mixed: AC-3* centromereglow type.Core granular glow type (AC-4-5*). | CD58(chr1:117078661 AT/A)-VUS<br>BANK1(chr4:102951224 GA/G) –VUS<br>SAMD9L(chr7:92762447 CA/C)-Likely pathogenic<br>LY96(chr8:74922341 CT/C) –VUS<br>DNASE1(chr16:3707747 G/A)-VUS<br>PTPN22(chr1:114381166 CT/C) –VUS<br>SLC5A11(chr16:24918057 CA/C) –VUS |
| S15 | f | SSc, chronic course, generalized, with skin lesions (dense swelling, “purse-string” mouth, sclerodactyly), blood vessels (Raynaud's syndrome), gastrointestinal tract (esophagitis), joints (arthritis) activity 1-2, Sjogren's syndrome (keratoconjunctivitis), Cushing’s syndrome. PAH. | | | 33 | 2 | 7,87 | 2560 | CENP-B, AC-3 | PTPN22(chr1:114381166 CT/C) –VUS<br>IRAK1(chrX:153278833 GCCCG/GCC)-VUS<br>SAMD9L(chr7:92764981 T/TT (reference sequencingTC))-LP<br>SAMD9L(chr7:92761606 GT/G)-LP<br>ABCC2(chr10:101559041 CA/C)-LP<br>AIRE(chr21:45711025 C/G)-VUS |
| S16 | m | Progressive SSc, subacute course, activity | 12 | D- | 15 | 3,79 | 5,5 | 160 | SS-A/60, SS-A/52, | CD58(chr1:117078661 AT/A)-VUS |

| | | | | | | | | | |
|---|---|---|---|---|---|---|---|---|---|
| | | 2, with skin lesions (dense edema, hyperpigmentation, induration), lungs (ILD, PAH), blood vessels (Raynaud's syndrome, telangiectasia), gastrointestinal tract(esophagitis), joints (arthritis), Sjogren's syndrome | penicillamin e500 мg | | | | | Scl-70. AC-21 Mitochondrial/reticular pattern. Coarse granular staining of filaments, extending throughout the cytoplasm. | BANK1(chr4:102951316 GA/G) – VUS<br>LY96(chr8:74922341 CT/C) –VUS<br>PTPN22(chr1:114381166 CT/C) – VUS<br>ARID5B(chr10:6385129 GA/G) – VUS<br>ITGAM(chr16:31289372 C/T) –VUS<br>SLC11A1(chr2:219259732 G/A) – VUS<br>IKZF3(chr17:37922552 T/C) –VUS |
| S19 | f | SSc, subacute course, activity 2, diffuse form activity 2, with lesions of the skin ((“purse-string” mouth), lungs (ILD, PAH), muscles, joints, heart, Raynaud's syndrome, Sjogren's syndrome | methotrexate 7,5 mg, mycophenolate mofetil 1000mg | 6 | 3,88 | 1,5 | 160 | SS-A/60, SS-A/52, U1-snRNP, RNP/Sm. AC-4. Core microgranular glow type. Fine, tiny granules are distributed throughout the nucleoplasm. | SAMD9L(chr7:92761606 GT/G)- Likely pathogenic<br>DNASE1(chr16:3707747 G/A)-VUS<br>REL(chr2:61149099 GT/G) -Likely pathogenic<br>PTPRC(chr1:198682102 AT/A) - Likely pathogenic<br>SMAD3(chr15:67477130 AT/A) – Pathogenic<br>CD40(chr20:44750489 GA/G) - Likely pathogenic<br>RBPJ(chr4:26417097 GT/G) –VUS<br>IL6ST(chr5:55265655 G/C) –VUS<br>CD5(chr11:60887035 C/G) –VUS<br>IL18(chr11:112014401 C/T) –VUS |
| S26 | f | SSc, chronic course, activity 2, with damage to the lungs (fibrosing alveolitis, respiratory failure 2), blood vessels (Raynaud's syndrome), joints (polyarthralgia), skin | | 17 | **14,6** | 6 | 640 | SS-A/52. Coregranularglowtype.(AC-4-5*).Granules are distributed throughout the nucleoplasm. | CD58(chr1:117078661 AT/A)-VUS<br>DNASE1(chr16:3707747 G/A)-VUS<br>PTPN22(chr1:114381166 CT/C) – VUS<br>PTPRC(chr1:198682102 AT/A) - Likely pathogenic<br>CD40(chr20:44750489 GA/G) - Likely pathogenic |

| |
|---|
| CTLA4(chr2:204736165 G/GT) - Likely pathogenic<br>IL6ST(chr5:55247857 GT/G) – Likely pathogenic<br>TNFSF4(chr1:173155905 TA/T)–VUS<br>PXK(chr3:58385095 T/C) –VUS<br>PRKCQ(chr10:6527154 AT/A) |

AC – anti-cell (ICAP, www.anapatterns.org); ANF – anti-nuclear factor (Anti-cellular antibodies, www.anapatterns.org); EScSG- The European Scleroderma Study Group activity index; f – female; ILD – interstitial lung disease; LP - likely pathogenic variant; m – male; P - pathogenic variant; PAH – pulmonary arterial hypertension; RSS - Rodnan skin score; SSc - Systemic sclerosis; VUS - variant of uncertain significance. Blue color means genes variations occurrence in both group of individuals.
